# Use of Geographic Information System Technology to Evaluate Health Disparities in Smoking Cessation Class Accessibility for Patients in Louisiana Public Hospitals

**DOI:** 10.3389/fpubh.2021.712635

**Published:** 2021-08-12

**Authors:** Tung Sung Tseng, Michael D. Celestin, Qingzhao Yu, Mirandy Li, Ting Luo, Sarah Moody-Thomas

**Affiliations:** ^1^Behavioral and Community Health Sciences, School of Public Health, Louisiana State University Health Sciences Center, New Orleans, LA, United States; ^2^Louisiana State University Health New Orleans, School of Medicine, New Orleans, LA, United States; ^3^Moores Cancer Center, School of Medicine, University of California San Diego, San Diego, CA, United States

**Keywords:** tobacco control, smoking cessation, geographic information system, distance, cancer control

## Abstract

Research has shown cigarette smoking is a major risk factors for many type of cancer or cancer prognosis. Tobacco related health disparities were addressed continually in cancer screening, diagnosis, treatment, prevention and control. The present study evaluated the health disparities in attendance of smoking cessation counseling classes for 4,826 patients scheduled to attend between 2005 and 2007. Of 3,781 (78.4%) patients with records to calculate the distance from their home domicile to counseling sites using Geographic Information System technology, 1,435 (38%) of smokers who attended counseling had shorter travel distances to counseling sites (11.6 miles, SD = 11.29) compared to non-attendees (13.4 miles, SD = 16.72). When the travel distance was >20 miles, the estimated odds of attending decreased with greater travel distance. Smokers who actually attended were more likely to be older, female, White, living in urban areas, and receiving free healthcare. After controlling for other socio-demographic factors, shorter distances were associated with greater class attendance, and individuals more likely to attend included those that lived closer to the counseling site and in urban settings, were female, White, commercially insured, and older than their counterparts. These findings have the potential to provide important insights for reducing health disparities for cancer prevention and control, and to improve shared decision making between providers and smokers.

## Introduction

Despite improvements in smoking cessation interventions, cigarette smoking continues to be the most preventable cause of morbidity and mortality in the United States (1). The 2019 United Health Foundation's report, *America's Health Rankings*, places Louisiana 49th in overall health, with smoking having the greatest negative impact on health (2). Tobacco use increases the risk of multiple cancers, such as lung, pancreas, bladder, stomach, and colon. In addition, continued tobacco use following a cancer diagnosis increases the risk of cancer recurrence, poor prognosis and adverse treatment-related outcomes (3). In Louisiana, males, ethnic minorities, persons aged 25 to 64, and those over 20 years old with less than a high school education smoke more than their counterparts (4). Between 2005 and 2009, more than 7,200 adults in Louisiana died annually due to smoking, resulting in more than 1.8 billion dollars of attributable health care expenditures in 2009 (4). Based on this data, public health advocates should identify barriers to accessing smoking cessation programs, to decrease the prevalence of smoking and smoking-related deaths in Louisiana.

Tobacco related health disparities were addressed continually in cancer screening, diagnosis, treatment, prevention and control. These health disparities often reflect a greater burden among vulnerable populations (5). A 2014 review identified three major barriers to smoking cessation amongst vulnerable populations (defined as disadvantaged populations facing lower income, cultural differences, and/or social exclusion): the use of smoking as stress management, high acceptability and prevalence of smoking within the community, and lack of support and access to smoking cessation programs (6). Such vulnerable populations are overrepresented in Louisiana. For example, in the 2010 population census, Louisiana had a greater percentages of persons living in poverty (19.7%) compared to the United States general population (12.3%) (7).

There are various types of smoking cessation interventions, including self-help materials, medication, telephone quit lines, and behavioral counseling (8). Behavioral counseling increases smoking quit attempts and rates of long-term smoking abstinence (9). A 2009 meta-analysis found that intensive interventions such as behavioral group counseling were more likely to promote smoking cessation compared to controls (10). A recent study has showed that telephone counseling would be effective under more real-world conditions (11). However, studies examining attendance of behavioral counseling have found low rates of participation due to factors such as low health literacy (12), high costs of attending counseling (13), and being a racial/ethnic minority (14).

Geographic Information System (GIS) technology has been utilized in a variety of health applications, such as modeling and mapping of disease location, monitoring disease spread, and assessing utilization of healthcare services (15). In the context of smoking, GIS has been employed in various applications, including analyzing demographic predictors of tobacco outlet density (16), assessing which neighborhoods were more likely to sell tobacco to minors (17), and monitoring tobacco industry billboard advertisements (18). However, few studies have utilized GIS to assess outcomes related to smoking cessation. In 2010, GIS analysis was employed to track the distribution and impact of a smoking cessation program in New York City (19). In this study, GIS was used to provide real-time visualization of participation in the cessation program. Findings showed that enrollment within the cessation program was higher in low-income, high-smoking prevalence neighborhoods, compared to high-income, high-smoking neighborhoods. Furthermore, GIS analysis was applied to assess the effectiveness of a message card campaign on compliance with the University of Kentucky's tobacco-free campus policy (20). GIS mapping software was used to display the location of cigarette butts, which were used as a measure of compliance. Additionally, GIS mapping has been utilized to demonstrate that tobacco outlet density is associated with knowledge of cigarette brand names (21).

These studies demonstrate the promising potential of GIS for assessing the effect of traveling distance on attendance of tobacco cessation counseling and smoking quit rates. Health outcomes related to distance can be analyzed via GIS in three main ways: travel time, road distance (distance between 2 points if traveling via roads), and map distance (direct distance on a map between 2 points) (22). A major assumption underlying studies of this type is that patients are more likely to use the health facility nearest to them; however, this may not always be the case in urban areas where there is a greater density of healthcare facilities (23). Conversely, in rural areas, patients are more likely to utilize the nearest health facility. Patients who reside in rural areas are less likely to quit smoking, in part due to a lack of local cessation programs (24). Furthermore, travel distance affects utilization of treatment, as demonstrated in regard to cancer treatments such as chemotherapy and radiation (25, 26). For example, a 2015 study used GIS to calculate the road distance between patients' residence and the nearest radiotherapy department and found less radiotherapy utilization with longer road distance from the patients' residence (26). Nevertheless, although people living in rural areas are more likely to travel longer distances to access smoking cessation programs, no studies have yet examined the effect of traveling distance on attendance of tobacco cessation counseling and smoking quit rates in urban and rural settings.

The Louisiana Tobacco Control Initiative (LA-TCI) is a statewide program that integrates evidence-based treatments into routine clinical practice within state hospitals of the LSU Health Care Services Division (LSU HCSD). Patients in these hospitals represent Louisiana's most medically vulnerable, with 49% being uninsured, and 77% being African-American (27). The LA-TCI provides free group behavioral counseling, which includes four consecutive 1-h sessions facilitated by certified tobacco treatment specialists (28). The initiative uses various methods, including GIS, to evaluate and improve cessation programs, visualize smoking prevalence, examine at-risk populations, and analyze trends. Previous LA-TCI studies include integrating evidence-based treatment of tobacco use into patient care practices (29) and demonstrating the utility of a health informatics system to optimize efforts to control tobacco use (30). The present investigation involved use of GIS to examine the effect of distance on attendance of smoking cessation class in a patient population with access to free counseling services provided by the LA-TCI.

## Methods

### Study Population

The study population included 4,824 LSU HCSD patients scheduled to attend counseling classes between 2005 and 2007. Of these, 3,910 (81.06%) had data available to calculate the distance between their residence and the referring hospital. Patients were excluded if (1) race reported was “Other,” (2) insurance status was missing, or (3) if they were listed as a “Prisoner.” Altogether, 3,781 patients were included. The LSU Health Sciences Center Institutional Review Board approved this research.

### Data Collection

The LA-TCI collects and reports data in the Cessation Management and Evaluation Database (CMED), a customized relational database developed to evaluate program delivery. At all facilities, CMED is used by TCI staff to identify opportunities for process improvements and to track program processes, such as patients referred, patients contacted, patients who participated in behavioral counseling, and prescription receipts for cessation medication.

### Measures

**Figure 1:**
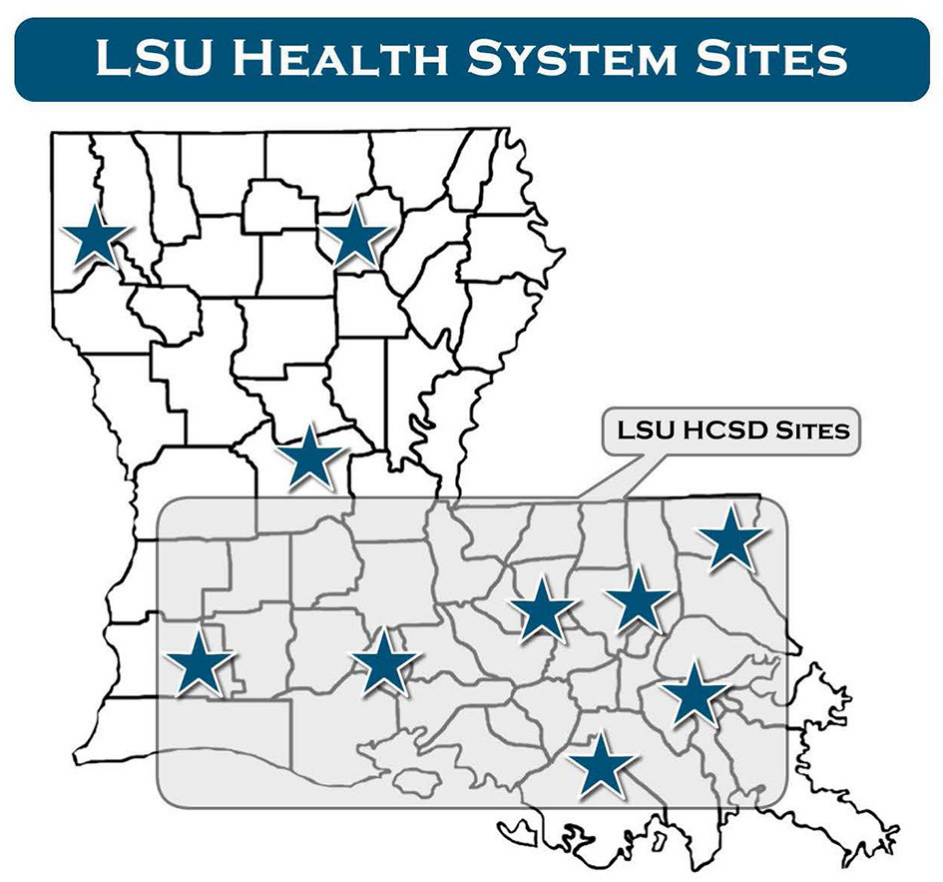
Louisiana Public Hospital system.

The outcome measure was class attendance, defined as scheduled patients who attended at least one 1-h group counseling session over the course of 4 weeks. The primary predictor variable was geographic distance, defined as the distance measured along the surface of the Earth. In other words, distances are defined by geographic coordinates in terms of latitude and longitude. With AreGIS software, patients' home addresses and counseling location were geocoded using geographical coordinates. Geocoding allows us to transform an address to a location on the earth's surface. We linked a table with patients' addresses, then we used Geocode to generate locations with geographic features, including attributes, and finally, we exported the data using R package for analysis. Covariates included age, gender (female, male), race (African American, White, Other), insurance status (Medicare, Medicaid, commercial, free care, and self-pay), and location (urban vs. rural hospital). We categorized hospitals as urban or rural based on population size according to the 2010 census. Urban hospitals included the Earl K. Long Medical Center (EKL) in Baton Rouge, the Walter O. Moss Medical Center (WOM) in Lake Charles, the Medical Center of Louisiana (MCL) in New Orleans, the University Medical Center (UMC) in Lafayette, and the Leonard J. Chabert Medical Center (LJC) in Houma, Louisiana (Figure 1). Rural hospitals included the Lallie Kemp Medical Center (LAK) in Independence and the Bogalusa Medical Center (BMC) in Bogalusa, Louisiana (Figure 1).

### Data Analysis

**Figure 2:**
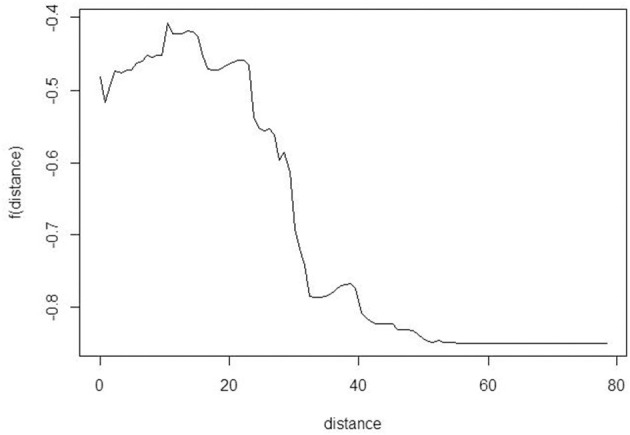
Partial dependence plot to explore the relationship between class attendance and distance to facility. The y-axis is the log odds of attending the smoking cessation class.

**Figure 3:**
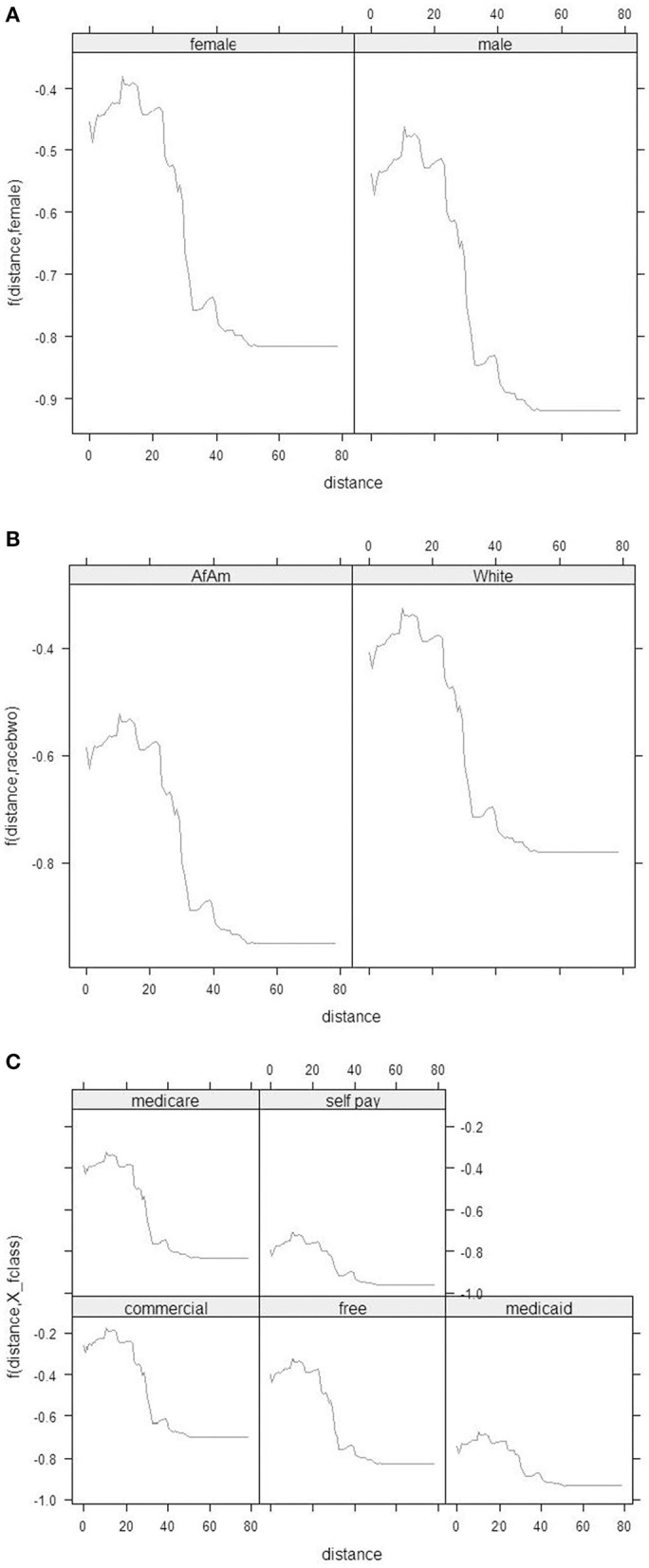
**(A)** Partial dependence plots between the distance (x-axis) and the log odds of attending the smoking cessation class by females and males separately. **(B)** Partial dependence plots between the distance (x-axis) and the log odds of attending the smoking cessation class by races. **(C)** Partial dependence plots between the distance (x-axis) and the log odds of attending the smoking cessation class by Payment Method.

**Figure 4:**
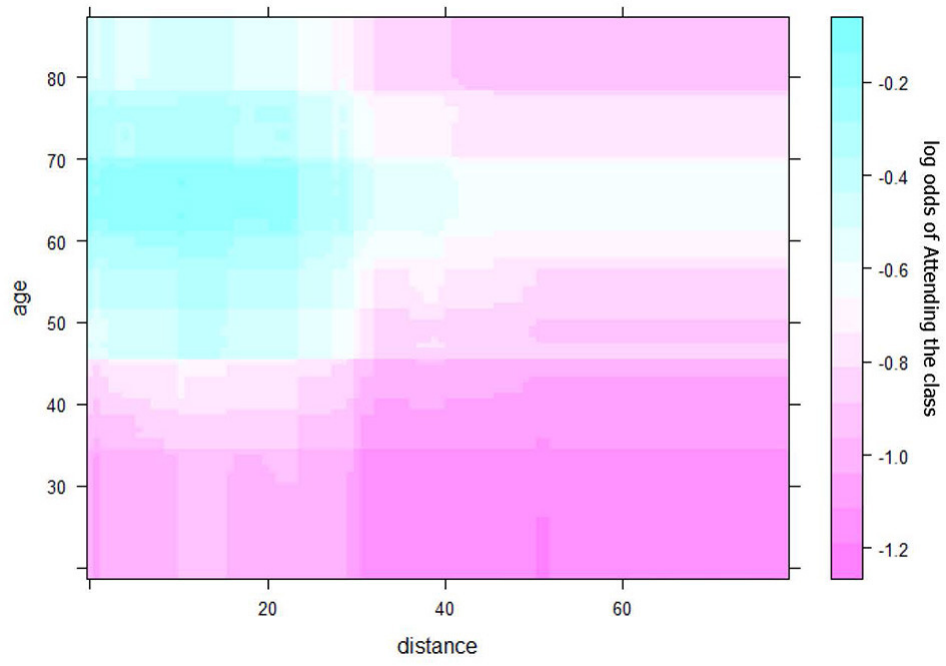
Joint effect of age and distance on class attendance.

Spearman's correlation, chi-square, and ANOVA determined the relationships between class attendance and distance and other risk factors individually. Multivariate logistic regressions jointly considered all risk factors and identified those associated with class attendance. Multiple Additive Regression Trees (MART) (31) illustrated the potential non-linear relationship and complex interactions of risk factors to explain the class attendance rate. All analyses were performed using R (4.0), except for geographic distance between the home address and counseling site for each patient, which was calculated using SAS and Excel. The MART analysis was performed using the R package gbm. When fitting the model, we use the out-of-bag samples to control the overfitting and set the shrinkage parameter at 0.001, and the total number of trees at 10,000. The maximum depth of variable interactions is set at 3 to avoid very complicated interactions. Partial dependence plots derived from the MART analysis were used to depict the relationships among variables. The partial dependence plot graphs the functional relationship between a small number of input variables and the outcome. In this paper, the outcome is the predicted log odds of attending the smoking cessation class. The plots show how the log odds (of attending class) changes with the distance to facilities (Figure 2), adjusting for other variables (Figures 3, 4).

## Results

Overall, 3,781 smokers who scheduled a group counseling class were included in this study. Of these, 38% (1,435) attended class. Table 1 provides demographic characteristics and class attendance for this sample. The probability of a smoker attending class was associated with the distance needed to travel to the counseling site. Smokers who attended class had shorter travel distances to counseling sites (11.6 miles, SD = 11.29) compared to those who scheduled but did not attend class (13.4 miles, SD = 16.72). When the travel distance was more than 15 miles (24 km), the estimated odds of attending class decreased with greater travel distance (Figure 2). Compared with patients who only scheduled class, smokers who actually attended class were more likely to be older (54.6 vs. 51.7%), female (69.9 vs. 64.9%), White (64.1 vs. 57.9%), living in urban areas (41.3 vs. 34.3%), and receiving free care (59.7 vs. 54.3%).

**Table 1:** Summary statistics classified by smoking cessation class attendance.

**Variables**	**Number of patients (%)**		*p*-value^**^
	**Scheduled only (*n* = 2,346)**	**Attended class (*n* = 1,435)**	
Age^*^	51.7 (11.35)	54.6 (10.5)	<0.001
Gender			0.002
Female	1,524 (64.96)	1,004 (69.97)	
Male	822 (35.04)	431 (30.03)	
Race			<0.001
African-American	986 (42.03)	514 (35.82)	
White	1,360 (57.97)	921 (64.18)	
Location			<0.001
Rural	1,539 (65.60)	841 (58.61)	
Urban	807 (34.34)	594 (41.39)	
Payer			<0.001
Medicaid	342 (14.58)	144 (10.03)	
Medicare	282 (12.02)	212 (14.77)	
Commercial	111 (4.73)	102 (7.11)	
Free care	1,274 (54.31)	857 (59.72)	
Self-pay	337 (14.36)	120 (8.36)	
Distance^*^	13.4 (16.72)	11.6 (11.29)	<0.001

* *Mean (SD) for continuous variables age and distances*.

** *To test the associations between each variable and the outcome (class attendance), we used ANOVA for the continuous variables and Chi-squared tests for the categorical variables*.

Results from logistic regression analyses revealed that shorter distances between home residence and counseling site were associated with higher class attendance rates, even after controlling for other socio-demographic factors (Table 2). Individuals more likely to attend counseling included those that lived closer to the counseling site (OR = 1.01, 95% CI = 1.01–1.02), lived in urban settings (OR = 1.51, 95%CI = 1.30–1.75), or were female (OR = 1.28, 95%CI = 1.11–1.49), White (OR = 1.66, 95% CI = 1.40–1.89), commercially insured (OR = 2.41, 95% CI = 1.72–3.44), or older (see OR = 1.03, 95% CI = 1.03–1.08) than their counterparts. There was no evidence for interactions between exposures (sex, race, insurance, and age) and outcome (class attendance).

**Table 2:** Logistic regression results on smoking cessation class attendance.

	**Comparison**	**Odds ratio (95% CI)**	***p*-value**
(Intercept)			<0.001
Distance	1 mile	0.99 (0.98, 0.99)	<0.001
Urban	Rural	1.51 (1.30, 1.75)	<0.001
Male	Female	0.78 (0.67, 0.90)	<0.001
White	Black	1.66 (1.40, 1.89)	<0.001
Age	1 year younger	1.03 (1.03, 1.08)	<0.001
Free	Commercial insurance	0.69 (0.52, 0.93)	0.013
Medicaid	Commercial insurance	0.46 (0.33, 0.64)	<0.001
Medicare	Commercial insurance	0.65 (0.46, 0.91)	0.012
Self-pay	Commercial insurance	0.41 (0.29, 0.58)	<0.001

*In the analysis, we included all variables that we analyzed to be significantly related with the outcome (class attendance, see Table 1)*.

Partial dependence plots (Figures 2, 3) show the visual relationship between class attendance and distance to facility. Figure 2 shows that the odds of attending class decreased dramatically if the smoker lived more than 15 miles (24 km) away. Similar patterns were observed for gender, age, and insurance type.

Figure 3 describes how distance from residence to hospital was related with class attendance. For distances within 20 miles (32 km), class attendance rates did not change regularly with greater distance. However, when the distance was >20 miles (32 km), the attendance rate decreased with greater distance. The distance-class attendance relationship changed by gender (Figure 3A), race (Figure 3B), and payment method (Figure 3C). The relationship was not changed significantly by the three variables as indicated by the almost parallel lines depicting the distance-class attendance relationship. Generally, at the same distance, males, African Americans, and patients with Medicaid or self-pay had lower class attendance rates compared with their counterparts.

Figure 4 shows the joint relationship of age and distance on class attendance. Smokers who were older and lived closer to counseling facilities were more likely to attend class. Although older smokers were more likely to attend class than younger smokers, age did not influence this relationship; hence, there was no interaction between age and distance on class attendance. In the analysis, age was considered a continuous variable. In general, people older than 60 had a higher average attendance rate when compared with younger smokers. Specifically, the attendance rate increased with age until about 65, and then decreased slightly.

## Discussion

For a patient population with access to free cessation counseling, this analysis examined the effect of distance between residence and counseling site on attendance of cessation counseling classes. Patients who were older, female, White, commercially insured, and with residences in urban areas were more likely to attend cessation counseling class than their counterparts. In addition, for those within a distance of 20 miles (32 km), class attendance rates did not change consistently with greater distance. However, as the distance increased beyond 20 miles (32 km), attendance rates decreased with greater distance. Although previous studies suggest that transportation difficulties and distance between residence and counseling site are associated with attendance for health education counseling (32), little is known about the relationship between distance and attendance of classes for smoking cessation counseling. Specifically, no studies have identified a cutoff point for how far is “too far” for smokers to utilize smoking cessation services. In addition, accessibility to health care service or smoking cessation class is a particular concern to reduce/eliminate health care disparities. A studies in South Africa also showed that distance plays a complex role in mediating health care utilization behavior. To reduce the distance that poor South Africans must travel to obtain health care in poorer areas will reduce inequality. Another study also showed that driving distance from the centroid of each census tract to the nearest CT facility in CT facility access has implications for lung cancer screening (LCS) implementation. Individuals in densely populated areas have relatively greater spatial access to CT facilities than those in sparsely populated tracts (33).

The results for age were consistent with previous reports showing that older patients may be more motivated to quit smoking and attend cessation counseling (34). Older patients are more likely to develop age-related medical illnesses that are exacerbated by smoking, and thus may be more likely to quit smoking in order to improve their health and/or longevity (35).

For this present population, class attendance was associated with gender and race. Females are more likely than males to participate in counseling-based smoking cessation activities (36, 37). Consistent with the present results, in a population of pediatric patients attending a weight management clinic, female patients were more likely to attend (38).

Black smokers are at greater risk for smoking cessation failure compared to their White counterparts (39). However, the factors that contribute to this disparity remain unclear. The present study found that White smokers were more likely to attend smoking cessation counseling classes compared to their Black counterparts, providing a possible explanation for why Black smokers are less likely than White smokers to quit.

Patients living in urban areas may not utilize the nearest health facility, as there may be multiple healthcare facilities within a reasonable distance (23). However, patients living in rural areas are indeed more likely to utilize the nearest health facilities. Patients living in rural areas are less likely to quit smoking in part due to a lack of local cessation programs (24). These results offer a potential explanation for our findings, which showed that smokers residing in rural areas were less likely to attend cessation classes than smokers residing in urban areas. Although rural cancer patients encounter substantial barriers to care, they more often report receiving timely care than urban patients. Recent studies also showed that Geographic distance differentially influences the initiation and completion of treatment among urban and rural cervical cancer patients (40).

Knowing the distance at which attendance of smoking cessation classes substantially decreases is important for inferring how far is “too far” for smokers to utilize smoking cessation services. The present study showed that, within 20 miles (32 km), class attendance rates did not change consistently with greater distance. However, as distance increased to more than 20 miles (32 km), attendance rates decreased sharply with increased distance. To our knowledge, this is the first study to identify a distance cutoff point for attendance of smoking cessation classes. The finding of a distance of 20 miles (32 km) is consistent with other studies. For example, in California, living within 20 miles (32 km) of receiving care was protective against mortality for patients with advanced-stage ovarian cancer (41). Also consistent with our results, another study found that racial and ethnic minorities residing within 20 miles (32 km) were less likely to receive care compared to Whites, and patients with low socioeconomic status (SES) were more likely to live farther away from treatment hospitals than their counterparts (42). Also similar to our results, colorectal cancer surgery patients living 30 km (18.6 miles) from a hospital possessed poorer survival prospects compared to patients who lived close by (43).

Identified distance points beyond which attendance significantly decreases show variations, depending on the main transportation type, type of treatment, and urban or rural location. For example, studies on hospital attendance in rural areas where patients generally reach health facilities by walking have found 3.0–3.5 km (1.9–2.2 miles) to be the distance where 50% of potential attendances are lost (44, 45). Although our finding of 20 miles (32 km) likely represents a driving distance, we cannot be certain what mode of transportation patients took to attend classes.

In the present population, the cost of attending class may have been problematic for smokers of lower SES. Using insurance status as a proxy for SES, we found that, compared to smokers of higher SES (commercial insurance), smokers of lower SES (self-pay, Medicaid, Medicare, or free care), were less likely to attend class. A previous study also found that privately insured patients were more likely to attend weight management class, and Gender and insurance status were the most significant predictors of class attendance (32), consistent with our results.

Results from the present study have limitations. First, since the study utilized a retrospective design, only associations could be determined. Further research is warranted to investigate the underlying etiology of these results. The study was also limited by selection bias, as we assessed only those smokers who were scheduled for and attended cessation counseling classes. Smokers who were not screened by hospital providers for smoking status, or were unable to access cessation services, may not be represented. In other words, the study focused only on smokers who were screened and scheduled for group counseling services. Moreover, since most smokers came from a low SES group, findings may not be translatable to the general population. Additionally, we measured geographic distance along the surface of the earth. With GIS technology, we geocoded patients' home address and class location using geographical coordinates. Thus, the distances measured may not reflect actual traveling time, due to traffic and road environments such as highways, mountains, and speed limits. However, previous studies suggest that distances estimated with GIS technology correlate with driving distances, and mean errors between the two are relatively small (46). Therefore, future studies should confirm the effect of travel time on class attendance in this population. Another limitation of this study is the time frame (2005–2007). However, with the exception of one clinic, the location of all clinics included in this study have remained the same since 2005–2007. The one clinic that changed location is less than half a mile away from the previous location, within the same zip code (previously LSU Interim Hospital, currently University Medical Center). Thus, the results of the current study may still reflect the current traveling distance for patients attending our group counseling classes.

## Conclusion

Among patients in Louisiana public hospitals, utilization of cessation counseling classes inversely related to the distance from residences to hospitals. Patients who were older, female, White, commercially insured, and with residences in urban areas were more likely to attend cessation counseling classes than their counterparts. To our knowledge, the present study is the first to identify a specific distance where smoking cessation class attendance significantly decreases. This study examined the effect of distance between residence and counseling site on attendance of cessation counseling classes, in a patient population with access to free classes for counseling. Patients who were older, female, White, commercially insured, and with residences in urban areas were more likely to attend cessation counseling classes than their counterparts. Therefore, smokers who live within 20 miles (32 km) of a smoking cessation class site should be considered a priority population for class recruitment. Further, when referring patients to smoking cessation classes, providers should take into account factors that limit patient participation, and consider offering alternative methods of obtaining smoking cessation resources. A distance of 20 miles (32 km) can be used to optimize locations for new smoking cessation programs. Using GIS tool is an efficient way to develop targeted interventions aimed at eliminating disparities in health for racial and ethnic minorities as well as other at-risk populations.

## Future Research

Future research should develop approaches for improving attendance of smoking cessation classes. Greater communication between patients and providers relating to barriers that patients face in obtaining smoking cessation resources is needed. Smoking cessation programs should consider providing more accessible smoking cessation counseling, such as mobile counseling in the community and telemedicine. Furthermore, studies should examine transportation methods and real driving times to smoking cessation resources. Further work is needed to identify access disparities of smoking cessation class to optimize smoking cessation service among eligible smokers for reducing health disparities for cancer prevention and control. Research in this field has the potential to guide tobacco control policies and to improve shared decision making between providers and smokers.

## Data Availability Statement

The original contributions presented in the study are included in the article/supplementary files, further inquiries can be directed to the corresponding author.

## Ethics Statement

The present study was reviewed and approved by the Institutional Review Board of Louisiana State University Health Sciences Center New Orleans. Written informed consent for participation was not required for this study in accordance with the national legislation and the institutional requirements.

## Author Contributions

TT and MC: conceptualization and data collection. TT and QY: methodology and data analysis. TT, MC, and ML: writing—original draft preparation. TL, ML, and SM-T: writing—review and editing. All authors have read and agreed to the published version of the manuscript.

## Conflict of Interest

The authors declare that the research was conducted in the absence of any commercial or financial relationships that could be construed as a potential conflict of interest.

## Publisher's Note

All claims expressed in this article are solely those of the authors and do not necessarily represent those of their affiliated organizations, or those of the publisher, the editors and the reviewers. Any product that may be evaluated in this article, or claim that may be made by its manufacturer, is not guaranteed or endorsed by the publisher.
